# Gastric Fundus Obstruction From Hiatal Hernia After Sleeve Gastrectomy: A Case Report

**DOI:** 10.7759/cureus.88018

**Published:** 2025-07-15

**Authors:** Amber Chen-Goodspeed, Angelina Kim, Ingrid Schmiederer, Joel Ricci-Gorbea

**Affiliations:** 1 Department of Surgery, New York-Presbyterian Queens, New York, USA

**Keywords:** bariatric, bariatric follow-up, bariatric surgery complications, gastric obstruction, hiatal hernia, postoperative complication, robotic surgery

## Abstract

Sleeve gastrectomy is currently the most common bariatric procedure in the United States, with over 750,000 cases performed between 2018 and 2022. While early complications such as hemorrhage and leaks are well documented, increasing data reveal a broader range of long-term complications, including stricture, gastroesophageal reflux, and hernias. We present a rare case of gastric sleeve herniation through a hiatal hernia (HH), resulting in gastric obstruction, a phenomenon not previously documented in the literature. A 78-year-old woman presented with abdominal pain, emesis, and anorexia. Imaging revealed incarceration of the gastric sleeve within a type III HH. Robotic-assisted hernia reduction, gastric resection, and HH repair were successfully performed. Postoperative recovery was uncomplicated, although a small recurrent hernia was noted at six months. This case underscores the importance of recognizing late postoperative complications and highlights robotic-assisted repair as a safe, effective approach even in acute care settings. This report also introduces a new long-term complication of sleeve gastrectomies that clinicians should include in their differential diagnosis. Further research on the benefits of screening for and repairing HHs during index sleeve gastrectomy may help prevent this complication.

## Introduction

Sleeve gastrectomy has become the most commonly performed bariatric surgery in the United States, accounting for over 65% of bariatric procedures, with more than 750,000 cases performed between 2018 and 2022 [1]. Its popularity stems from its relative technical simplicity compared to other bariatric procedures and its effectiveness in achieving significant and sustained weight loss [2]. The procedure involves resecting the stomach parallel to the lesser curvature, forming a narrow gastric sleeve. This results in earlier satiety due to reduced gastric volume and suppressed ghrelin production.

Commonly studied early postoperative complications include hemorrhage, infection, and gastric leaks. Long-term complications include stricture formation, gastroesophageal reflux disease (GERD), and nutritional deficiencies [2,3]. As more long-term follow-up data become available, additional complications are being identified.

This case report describes a rare long-term complication: herniation of the gastric sleeve through a hiatal hernia (HH), resulting in gastric obstruction, occurring three years after sleeve gastrectomy. To our knowledge, this specific complication has not been previously reported in the medical literature. The development of an HH is thought to result from increased intraabdominal pressure and laxity of the phrenoesophageal ligament, which allows the gastroesophageal junction or a portion of the stomach to herniate into the thoracic cavity [4]. Diagnosis of HHs after bariatric surgery can be challenging, as patients can present with nonspecific symptoms such as bloating and nausea. While HHs after sleeve gastrectomies have been reported, current publications primarily focus on their association with GERD, not obstruction [5-8].

We present the case of a 78-year-old woman who developed gastric obstruction secondary to herniation of her gastric sleeve into her mediastinum three years after her laparoscopic sleeve gastrectomy. This rare but serious complication was successfully managed with a minimally invasive repair technique in the acute setting.

This article was previously presented as a meeting abstract at the ASMBS Scientific Meeting on June 27, 2023.

## Case presentation

A 78-year-old woman with a history of laparoscopic sleeve gastrectomy in 2019 presented to the emergency department in 2022 with severe abdominal pain, dysphagia, anorexia, and two days of constipation. After her sleeve gastrectomy, she reported a total body weight loss of 29% and excess body weight loss of 60%, resulting in a decrease in BMI from 46 to 32. She was a former smoker and no longer required continuous positive airway pressure (CPAP) therapy for sleep apnea after her initial bariatric surgery. Although her initial weight loss was beneficial, she noted a plateau in her weight loss over time.

For one month prior to presentation, she experienced intermittent epigastric pain radiating to the back after eating, with episodes lasting from several minutes to multiple hours. Her pain progressively worsened and became acutely severe, prompting her visit to the emergency department. Her associated symptoms at the time of presentation included emesis, gastric reflux, and subjective fever. She denied nausea at the time of presentation.

On arrival, she was febrile. On physical exam, her abdomen was soft, non-distended, and non-tender to palpation with no peritoneal signs. Laboratory studies revealed an acute kidney injury, leukocytosis with left shift, and hyperlactatemia (Table 1).

**Table 1:** Pertinent laboratory results at the time of patient presentation

Result Name	Patient Value	Normal Range
White Blood Cell (WBC) Count (K/uL)	13.9	4.8-10.8
Neutrophil % (%)	84.7	37.0-80.0
Lactate (mmol/L)	2.3	0.5-1.6
Creatinine (mg/dL)	1.20	0.51-0.95

**Figure 1:**
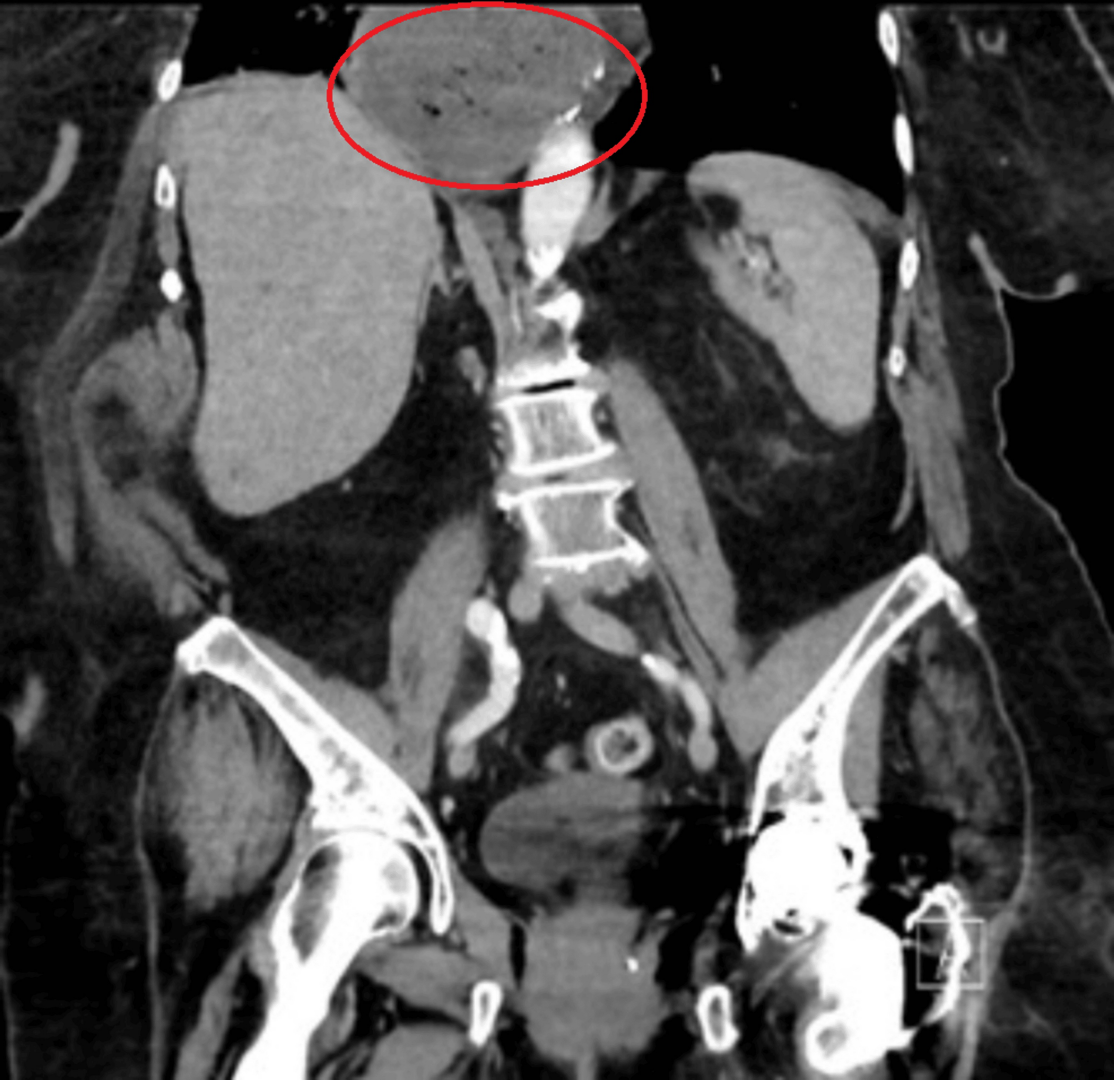
CT abdomen and pelvis demonstrating gastric sleeve herniated into the mediastinum Red circle indicates the gastric sleeve within the hiatal hernia.

A CT scan demonstrated a 9 cm × 6.5 cm stomach above the diaphragmatic hiatus containing heterogeneous material and a small amount of gas (Figure 1).

The patient was admitted for gastric obstruction and started on fluids for dehydration and empiric antibiotics due to concern for aspiration pneumonia. Nasogastric tube placement was attempted but was unsuccessful. Due to the inability to decompress the stomach, she was taken to the operating room for a diagnostic laparoscopy.

A 12 mm optical trocar was inserted via a transverse incision to the right of the umbilicus. Intraoperative inspection revealed a type III HH, with the majority of her gastric sleeve incarcerated in the mediastinum. Three 8 mm ports and a Nathanson liver retractor were placed, and the robot was docked.

**Figure 2:**
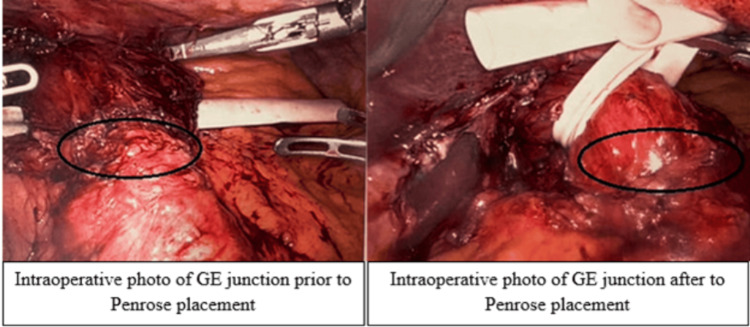
Intraoperative placement of Penrose drain encircling the gastroesophageal junction Gastroesophageal junction marked by black circle on both pictures.

The stomach was identified, and a window was created in the greater omentum to access the lesser sac. Extensive lysis of adhesions was performed. The mediastinum was entered by dissecting along the crura into the retroesophageal space. A Penrose drain was passed around the esophagus, and gentle traction on the Penrose drain facilitated successful reduction of the stomach into the abdominal cavity (Figure 2). The hernia sac was excised, and 3 cm of tension-free intraabdominal esophagus was ensured. The diaphragmatic hiatus was closed by approximating the right and left crura using a non-absorbable barbed suture. The Penrose drain was removed. 

No fundopexy was performed due to the poor quality of the chronically obstructed, macerated gastric fundus and prior partial fundus resection during the original sleeve gastrectomy. The devascularized portion of the stomach was resected using a robotic stapler. A leak test was negative. A Jackson-Pratt (JP) drain was placed near the gastric staple line. All ports were closed in the standard fashion, and the patient was extubated.

Postoperatively, the patient was monitored in the surgical step-down unit. On postoperative day one, bowel function returned, and an upper GI series confirmed patency of the gastroesophageal junction. The JP drain was removed, and a clear liquid diet was initiated. On postoperative day two, the patient was advanced to a regular diet. She was discharged on postoperative day four.

**Figure 3:**
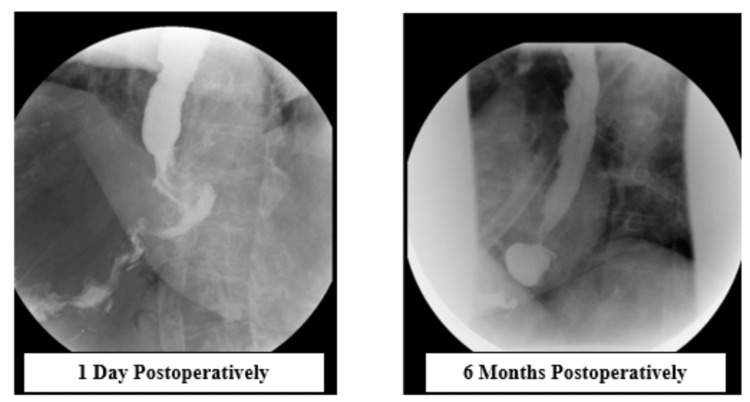
Upper gastrointestinal (UGI) series at postoperative day one and postoperative month six Image on the left demonstrates the patent gastroesophageal (GE) junction following operative repair with the absence of hiatal hernia; image on the right demonstrates a de novo hiatal hernia following hiatal hernia repair.

At two-week and eight-week follow-ups, the patient was recovering well. At six months, she reported mild discomfort and bloating with an 18-pound weight gain. A repeat upper GI series demonstrated a small recurrent HH (Figure 3). She continues to follow up with the bariatric surgery team.

## Discussion

This case highlights a rare but important complication not previously documented in the literature: gastric obstruction secondary to an HH after sleeve gastrectomy. Clinicians should consider this diagnosis in patients presenting with oral intake intolerance, emesis, and abdominal pain after bariatric surgery. The diagnostic workup may include esophagram, computed tomography (CT), and potentially esophagogastroduodenoscopy (EGD). Obstruction due to herniation of the gastric sleeve above the diaphragm presents acutely and warrants urgent surgical intervention.

Gastric obstruction typically results from mechanical blockage of the pylorus, stomach, or duodenum [9]. While malignancy is a common cause, benign etiologies such as peptic ulcer disease, bezoars, and volvulus remain on the differential. Symptoms often include early satiety, nausea, vomiting, and epigastric pain. Management depends on the underlying cause and may involve medical, endoscopic, or surgical interventions [9].

HHs occur when intraabdominal contents herniate through the esophageal hiatus, typically due to laxity of the phrenoesophageal ligament [4]. This herniation can compromise the lower esophageal sphincter (LES), increasing the risk of GERD. Risk factors for HH formation include obesity, pregnancy, and obstructive pulmonary disease. Although many HHs are asymptomatic, symptoms may include GERD, regurgitation, or chronic cough. HHs are classified into four types. Type I involves sliding of the gastroesophageal junction above the diaphragm. Type II is a paraesophageal hernia with a small portion of stomach above the diaphragm, but with the gastroesophageal junction below the diaphragm. Type III combines I and II. Type IV includes herniation of other abdominal organs [4]. Diagnosis is achieved through endoscopy, manometry, pH testing, or esophagram. Treatment ranges from lifestyle changes and medical therapy to surgical repair [4].

Primary HH refers to hernias present before sleeve gastrectomy, while de novo HH refers to those that develop postoperatively. Standard surgical treatment for primary HH in patients not undergoing concurrent bariatric surgery is a posterior crural repair with fundoplication to restore the anatomic relationship between the esophagus and diaphragm and reinforce the LES [10].

Fundoplication after sleeve gastrectomy is technically complex due to significantly reduced gastric fundus size after gastrectomy [11]. To account for this altered anatomy, alternative techniques must be considered when repairing HH during initial sleeve gastrectomy. Castagneto-Gissey et al. compared the efficacy of sleeve gastrectomy with concurrent HH repair versus sleeve gastrectomy with fundoplication. They found that sleeve gastrectomy with fundoplication achieved superior GERD remission (5% vs. 20.4%, p < 0.001) compared to sleeve gastrectomy with primary hiatus repair, but at the cost of higher complication rates (gastric perforation 3.1% vs. 0%) and mortality rates (0.5% vs. 0%) [11]. The authors highlighted that fundoplication utilizing a gastric sleeve was technically difficult, likely contributing to the higher complication rates [11].

Hutopila et al. proposed reconstruction of the phreno-esophageal ligament (R-PEL) as a novel technique to prevent intrathoracic sleeve migration and GERD [12]. R-PEL involves suturing the esophagus to the diaphragm at the original ligament insertion points, effectively anchoring the GE junction [12]. In their 2023 study, R-PEL reduced intrathoracic sleeve migration from 50.7% to 8.7% and significantly improved GERD symptoms compared to crural repair alone [12]. This technique may be technically simpler and safer than fundoplication after sleeve gastrectomy; however, the two techniques have not been directly compared.

While several techniques are available for concurrent HH repair, there is no current consensus on whether an HH should be repaired at the time of the initial bariatric surgery. For instance, in a study of 2,623 bariatric patients, only 3.2% of those with HHs diagnosed at the time of initial bariatric surgery underwent concurrent HH repair [13]. However, concurrent HH repair is becoming more common [14]. A larger study involving 222,320 patients found no increase in 30-day complications with concurrent hernia repair, though operative times and readmission rates were higher [15].

The development of de novo HHs is likely due to postoperative anatomical changes such as disruption of the phrenoesophageal ligament, altered crural support, and loss of the angle of His [16,12]. These changes can predispose the GE junction to migrate into the thoracic cavity. Saba et al. reported that the incidence of de novo HH after sleeve gastrectomy in patients without primary HH was as high as 80% at 18 months postoperatively [16]. De novo HHs often present similarly to primary HHs, with GERD, esophagitis, and increased proton pump inhibitor (PPI) dependence [16]. They can also demonstrate more nonspecific symptoms as encompassed by Golas et al.'s acronym “BARF”: bloating, abdominal pain, regurgitation, and food intolerance/dysphagia [17].

The current gold standard management of de novo HH causing GERD or other symptoms mirrors that of type I and II primary HH: lifestyle modification followed by PPI. If symptoms persist, surgical management includes elective HH reduction and cural repair with the option of conversion to Roux-en-Y-gastric bypass (RYGB) if BMI remains over 35 [18]. Surgical reintervention is reserved for persistent or severe symptoms, as reoperations carry increased morbidity [19]. However, in the cases of acute gastric obstruction, as in this report, the risk of gastric necrosis necessitates emergent operative intervention, bypassing the usual medical-to-surgical pathway.

This report demonstrates that robotic-assisted HH repair and reduction are feasible and effective in the emergent setting. Minimally invasive surgical approaches are associated with reduced postoperative pain, lower infection rates, and shorter hospital stays [20]. The robotic platform provides enhanced visualization of the diaphragmatic hiatus and the herniated gastric sleeve, facilitating a safe and thorough repair.

Further research is needed to determine whether RYGB or sleeve gastrectomy with concurrent HH repair is more appropriate for patients with HHs at the time of initial bariatric surgery. Additionally, studies comparing techniques for HH repair in the sleeve gastrectomy population, both for primary and de novo hernias, are warranted.

## Conclusions

This report demonstrates that gastric obstruction secondary to gastric sleeve herniation should be considered in patients with early satiety, bloating, and food regurgitation after sleeve gastrectomy. Prompt diagnosis with CT imaging or esophagram, along with attempts at nasogastric decompression, is an essential first step in management. Robotic-assisted reduction of the herniated gastric sleeve with concurrent HH repair offers a safe and effective surgical option even in the acute setting. Given the potential for this rare but serious complication, there is a growing need for standardized guidelines regarding the management of HHs identified at the time of initial bariatric surgery. Large-scale studies comparing outcomes of sleeve gastrectomy alone versus sleeve gastrectomy with each method of concurrent hernia repair, as well as comparisons with RYGB, are needed to inform best practices and optimize patient outcomes.
